# Targeting NEDDylation as a Novel Approach to Improve the Treatment of Head and Neck Cancer

**DOI:** 10.3390/cancers13133250

**Published:** 2021-06-29

**Authors:** Trace M. Jones, Jennifer S. Carew, Julie E. Bauman, Steffan T. Nawrocki

**Affiliations:** Department of Medicine, The University of Arizona Cancer Center, Tucson, AZ 85724, USA; tracejones@email.arizona.edu (T.M.J.); jcarew@arizona.edu (J.S.C.); jebauman@arizona.edu (J.E.B.)

**Keywords:** head and neck cancer, NEDD8, NEDDylation, Pevonedistat, MLN4924

## Abstract

**Simple Summary:**

Head and neck cancer is a complex and heterogeneous disease that affects nearly 900,000 individuals every year. Despite this, very few treatment options exist, particularly for patients diagnosed with late-stage disease. Currently approved therapies for head and neck tumors display limited anticancer activity, which highlights the need for more effective treatment options. In this review, we discuss an exciting new class of drugs that inhibit the NEDDylation pathway. NEDDylation is a protein modification pathway which affects the appropriate degradation of a wide variety of targets. NEDDylation is often hyperactivated in head and neck cancers and, thus, makes for a potential therapeutic target. To date, several compounds have been developed to block NEDDylation including pevonedistat (MLN4924) and TAS4464. Inhibition of NEDDylation has shown promising results in a variety of head and neck cancer cell lines, animal models, and early stage clinical trials. This review will summarize the mechanisms of action of existing NEDDylation inhibitors and their status in clinical development.

**Abstract:**

Head and neck cancer is diagnosed in nearly 900,000 new patients worldwide each year. Despite this alarming number, patient outcomes, particularly for those diagnosed with late-stage and human papillomavirus (HPV)-negative disease, have only marginally improved in the last three decades. New therapeutics that target novel pathways are desperately needed. NEDDylation is a key cellular process by which NEDD8 proteins are conjugated to substrate proteins in order to modulate their function. NEDDylation is closely tied to appropriate protein degradation, particularly proteins involved in cell cycle regulation, DNA damage repair, and cellular stress response. Components of the NEDDylation pathway are frequently overexpressed or hyperactivated in many cancer types including head and neck cancer, which contribute to disease progression and drug resistance. Therefore, targeting NEDDylation could have a major impact for malignancies with alterations in the pathway, and this has already been demonstrated in preclinical studies and clinical trials. Here, we will survey the mechanisms by which aberrant NEDDylation contributes to disease pathogenesis and discuss the potential clinical implications of inhibiting NEDDylation as a novel approach for the treatment of head and neck cancer.

## 1. Head and Neck Cancer

Head and neck cancer is a broad term describing malignancies whose origin can be traced to the upper aerodigestive tract. Worldwide, 890,000 new cases were diagnosed in 2018, accounting for 3% of all cancer diagnoses [1]. The vast majority of head and neck cancers are of epithelial origin and can be classified as squamous cell carcinomas. These tumors arise in the mucosal surfaces of the oral cavity, pharynx, larynx, and paranasal sinuses [2]. Due to this variability of primary sites, head and neck cancer is highly heterogenous. As a consequence, clinical management must be tailored to the malignancy’s tumor, node, metastases (TNM) staging, anatomical location, and viral vs. tobacco-related carcinogenesis.

One etiologic distinction of major clinical importance is the human papillomavirus (HPV) status of the tumor. HPV type 16 is the predominant strain responsible for carcinogenesis in the head and neck [3,4]. For reasons which are not entirely clear, HPV+ tumors have a strong predilection to occur in the oropharynx, but rarely in other adjacent sites [4,5]. More than 70% of oropharyngeal cancers in the United States are positive for the HPV16 genome [4]. Patients with HPV+ tumors are generally younger and have fewer comorbidities than their HPV- counterparts, including fewer pack-years of tobacco exposure. In addition, HPV+ tumors respond favorably to surgical resection, radiotherapy, and chemotherapy. Due to these factors, patients with HPV+ disease have an overall survival rate in excess of 80% [6].

In contrast, patients who present with HPV- disease have a much less favorable prognosis, with a 5-year survival rate of approximately 50% [6,7]. This is, in part, due to approximately two-thirds of patients presenting with advanced disease [8]. Additionally, HPV- tumors are highly associated with chronic alcohol and tobacco abuse [9]. Consequently, many patients with HPV- head and neck cancer have other comorbidities that compromise successful clinical management. Finally, HPV- tumors tend to be quite aggressive. At least 50% of patients will experience a local or distant recurrence within two years of therapy [10]. Due to the unsatisfactory survival rates, as well as the aggressive nature of HPV- head and neck cancers, this review will focus on a class of exciting new therapeutics aimed to combat HPV- head and neck cancer.

## 2. Current Therapeutic Strategies

Due to etiologic and anatomic heterogeneity, the treatment plans for patients with head and neck cancer require a tailored, multimodal approach including surgery, radiation therapy (RT), and systemic therapy. Currently indicated systemic therapies in head and neck cancer include the cytotoxic chemotherapies cisplatin, docetaxel, the anti-metabolite fluorouracil (5-FU); the anti-epidermal growth factor receptor (EGFR) monoclonal antibody (mAb) cetuximab; and the anti-programmed death receptor-1 (PD-1) mAbs pembrolizumab and nivolumab. For patients presenting at an early stage (I or II), surgery to remove the primary mass can be curative as a single modality and is often the preferred strategy. If surgical resection is not viable due to morbidity concerns, definitive RT is often a highly effective alternative. Surgery or RT alone yield a 70–90% long-term survival rate in patients with early-stage disease [11]. Patients with locally advanced or distant metastatic disease (stage III or IV) have a significantly worse prognosis. Even with the advent of EGFR-targeted and anti-PD-1 mAb therapies, patients with advanced disease still have a < 50% overall survival rate. This dismal outcome has remained nearly unchanged over the past four decades [12]. Surgical resection is still the preferred first option for locally advanced malignancies of the oral cavity, followed by adjuvant RT and concurrent cisplatin as indicated by pathologic features. However, due to the important human functions of the pharynx and larynx, including speech and swallowing, organ preservation approaches combining definitive RT with cisplatin or cetuximab are an accepted standard of care. The standard of care nonsurgical regimen established in the Radiation Therapy Oncology Group (RTOG) 91–11 trial is high-dose cisplatin (100 mg per square meter of body-surface area, given every 21 days) administered concurrently with RT [13]. In addition, induction chemotherapy with 5-FU and cisplatin, with or without docetaxel, has shown some benefit when preceding irradiation [14]. Despite modest success involving these DNA damaging agents, substantial hurdles still remain. High toxicity is common with platinum-based regimens, particularly in patients who are elderly or have poor performance status. Carboplatin can be substituted for cisplatin in order to reduce toxicities, however this compromises therapeutic efficacy in the curative setting [15]. Although induction therapy with cisplatin doublets or triplets yields a high response rate, this approach does not improve survival compared to definitive cisplatin-RT and has fallen out of favor except in specific clinical circumstances, such as T4 tumor stage [16]. There is an urgent medical need for development of compounds that are more effective and with fewer adverse effects.

## 3. NEDDylation as a Therapeutic Target

The dysregulation of protein homeostasis is a prominent feature in many forms of cancer including head and neck cancer [17]. Multiple pathways can have an impact on the degradation of cellular proteins. One such pathway that has been shown to be hyperactivated in cancer is NEDDylation. Neural precursor cell expressed, developmentally-downregulated 8 (NEDD8) is a ubiquitin-like protein that is conjugated to target proteins in order to modulate their function [18]. The NEDDylation cascade is controlled by a series of enzymes in a fashion similar to ubiquitination. First, a heterodimer of ubiquitin-like modifier activating enzyme 3 (UBA3) and NEDD8 activating enzyme E1 regulatory subunit (APPBP1) forms, resulting in an active NEDD8-Activating Enzyme (NAE) [19]. NAE acts as the E1 ligase in the NEDDylation cascade and is, thus, responsible for initiating the NEDDylation reaction. NAE binds and activates free NEDD8 through covalent interactions [20,21]. NEDD8 proteins are then transferred to one of two E2 ligases, NEDD8 conjugating enzyme (UBC12) and NEDD8-conjugating enzyme 2 (UBE2F), before being shuttled to one of many E3 NEDD8 ligases [22]. These E3 ligases provide the specificity for substrates, much like in the ubiquitin pathway.

**Figure 1:**
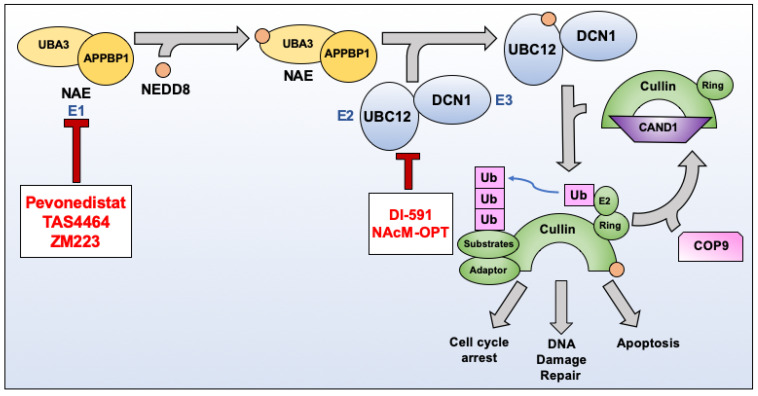
Representative model of the compounds which target the NEDDylation pathway. PEV, TAS4464, and ZM223 all target NAE. PEV inhibits NEDDylation by forming a covalent NEDD8-PEV adduct within the NAE active site to block enzymatic activity. DI-591 and NAcM-OPT disrupt the binding of DCN1 with UBC12, thus inhibiting the NEDDylation of a subset of Cullin-RING ligases. Abbreviations: NAE—NEDD8 activating enzyme; UBA3—Ubiquitin-like modifier activating enzyme 3; APPBP1—NEDD8 activating enzyme E1 regulatory subunit; UBC12—NEDD8 conjugating enzyme Ubc12; DCN1—Defective in Cullin NEDDylation 1; Ub—Ubiquitin; COP9—Constitutive photomorphogenesis 9; CAND1—Cullin-associated NEDD8-dissociated protein 1.

**Figure 2:**
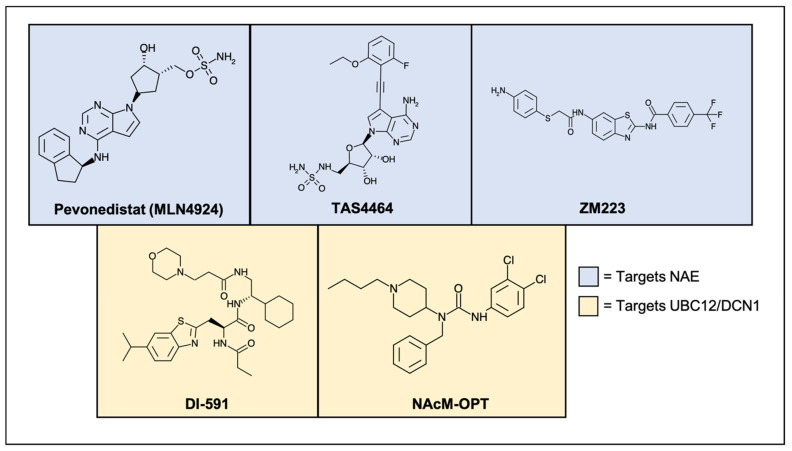
Chemical structures of molecules that inhibit various proteins in the NEDDylation pathway. PEV and TAS4464 are sulfonamide-based NAE inhibitors which have entered clinical trials. PEV has displayed promising clinical activity in a variety of cancer types. ZM223 does not contain a sulfonamide group and inhibits NAE through different covalent interactions. DI-591 and NAcM-OPT competitively bind and inhibit the interaction of UBC12 and DCN1, thus prohibiting downstream NEDD8 conjugation.

The most well understood of the NEDD8 E3 ligases are RING-box protein 1 (RBX1) and RING-box protein 2 (RBX2). RBX1 and RBX2 interact with the E2 ligases, UBC12 and UBE2F, respectively, in order to conjugate NEDD8 to members of the Cullin-RING ligase (CRL) family [22,23]. The CRLs are a group of ubiquitin ligases that require the addition of a C-terminal NEDD8 modification in order to become activated [24,25,26]. The constitutive photomorphogenesis 9 (COP9) signalosome is a protein complex capable of interacting with NEDDylated CRLs and removing the NEDD8 modification, thereby reversing the activity of the NEDDylation cascade and acting as an “off” switch to the Cullins [27]. CRLs function as E3 ubiquitin ligases in the ubiquitin-proteasome protein degradation pathway [25,28]. There are a total of eight known Cullin proteins, and they are responsible for regulating the degradation of hundreds of cellular proteins. A list of the Cullins, as well as important targets, can be found in Table 1. NEDDylation of CRLs causes the displacement of the inhibitory protein Cullin-associated NEDD8-dissociated protein 1 (CAND1) [29,30]. This allows for a conformational shift, resulting in CRL activation and subsequent ubiquitination of substrates. CRL target proteins include many cell cycle regulators, such as cyclin-dependent kinase inhibitor 1 (p21, Cip1, Waf1), cyclin-dependent kinase inhibitor 1b (p27, Kip1), and Wee1 G2 checkpoint kinase (Wee1), as well as proteins involved in chromatin remodeling, DNA damage repair, and apoptotic signaling [31,32,33,34,35,36]. Therefore, a hyperactivated NEDDylation pathway as seen in cases of head and neck cancer leads to the dysregulation of CRL target protein degradation and dysfunction of the aforementioned pathways [37]. A schematic of this process can be found in Figure 1. It is due to both the overexpression of pathway components as well as their involvement with key oncogenic controls that make NEDDylation an attractive therapeutic target. This review will highlight the inhibitory compounds that are currently in preclinical and clinical development, with a particular focus on their use in head and neck cancer where applicable. A list of the compounds present in this review can be found in Table 2. The chemical structures of these novel compounds are displayed in Figure 2.

### 3.1. Pevonedistat (MLN4924)

Pevonedistat (PEV) is a first-in-class small molecule inhibitor of NAE developed by Millennium/Takeda Pharmaceuticals (Tokyo, Japan) [49]. PEV is a highly selective adenosine mimetic that forms a covalent adduct with NEDD8 and irreversibly binds to the active site of NAE, thereby impairing any further conjugation of NEDD8 to downstream substrates [50]. Various studies have demonstrated that the inhibition of NEDDylation by PEV has a strong inhibitory effect on CRLs [51,52]. This inhibition results in the stabilization of a large variety of CRL targets, which in turn produces an assortment of phenotypic defects. PEV treatment often results in S phase stalling and DNA re-replication as a result of the stabilization of chromatin licensing and DNA replication factor 1 (CDT1), an essential component of the DNA pre-replication complex [49,53,72]. As a result, a large proportion of cells exhibit greater than 4N genomic content when analyzed via propidium iodide-fluorescent activated cell sorting (PI-FACS) [53]. This phenotype is thought to lead to apoptosis as the cell is no longer viable. In addition, cell cycle regulators such as p21, p27, and Wee1, are also highly stabilized following treatment with PEV. This results in a strong suppression of proliferation. To date, PEV has been studied in a variety of tumor models with ovarian, colorectal, renal, and acute myeloid leukemia (AML) responding particularly well to the inhibitor [52,53,54,55,63]. Given the importance of NEDDylation to cancer development and progression, PEV has demonstrated promising anticancer activity across a wide range of cancer types.

Due to the previously described need for novel therapeutics, head and neck cancer is one particular cancer type in which PEV is currently being investigated. PEV has been shown to enhance the effects of RT in various head and neck squamous cell carcinoma (HNSCC) cell lines. Cullin-4 is a CRL responsible for complexing with denticleless protein homolog (CDT2) and subsequently targeting CDT1 for proteasomal degradation [73]. Inhibition of Cullin-4 via PEV drives the DNA re-replication effect that was discussed above. DNA re-replication, with the resulting genomic instability, was shown to be the mechanism by which PEV sensitizes otherwise resistant cells to ionizing radiation [59]. It was demonstrated that pre-treatment of FaDu and Cal27 HNSCC cells with 20–80 nM of PEV resulted in significantly lower survival rates following 2–4 Grays (Gy) of RT than non-pre-treated cells. In addition, cells that were subjected to both RT and PEV exhibited higher levels of phosphorylated checkpoint kinase 1 (p-Chk1), phosphorylated checkpoint kinase 2 (p-Chk2), and gamma H2A histone family member X (γH2AX), markers indicative of substantial DNA damage. In vivo, the combination of PEV and RT resulted in significantly lower Cal27 xenograft tumor burden in mice and was well tolerated. Given the importance of RT in many patient treatment plans, utilization of PEV as a radiosensitizer could prove a valuable tool in the clinical setting.

It was also recently discovered that nasopharyngeal tumors and cell lines exhibit a high baseline level of NEDD8 protein, and this elevated expression correlates with poor patient outcomes [37]. Xie et al. [37] analyzed a total of 197 patient specimens for NEDD8 expression using immunohistochemistry approaches. Specimens were then divided into low and high expression groups. Median overall survival was significantly shorter in the high expression group (*p* = 0.002). However, it should be noted that nasopharyngeal tumors from Southeast Asia are frequently caused by Epstein–Barr virus (EBV) and generally respond positively to chemotherapy. Interestingly, the authors of this study also observed that knockdown of NEDD8 in nasopharyngeal cancer cell lines resulted in decreased cell viability and this effect was made more pronounced by the introduction of standard of care agents such as cisplatin. This suggests that PEV may improve the efficacy of existing HNSCC treatments. PEV was administered to mice bearing S18 xenograft tumors and reduced tumor volume, providing evidence of its bioavailability and antitumor properties. Other studies have demonstrated that the anticancer effects of PEV may be related to the upregulation of Phorbol-12-myristate-13-acetate-induced protein 1 (NOXA) and bcl-2-like protein 11 (BIM), two bcl-2 homology 3 domain (BH3)-only proteins that contribute to the activation of apoptosis [56,57]. The induction of these pro-apoptotic factors may explain the synergistic interaction of PEV and tumor necrosis factor-related apoptosis-inducing ligand (TRAIL), a death receptor activating ligand, in HNSCC cells [58].

Based on the promising anticancer efficacy that has been observed in many preclinical studies, PEV has been evaluated in a variety of clinical trials. Initial phase I trials of PEV were conducted in patients with AML. PEV was investigated as both a single-agent in a refractory population and as an adjuvant to azacitidine therapy in treatment-naïve patients [60,61]. The maximum tolerated dose (MTD) of PEV as a single agent was reported as 59–83 mg/m^2^, depending on administration schedule. However, in combination with azacitidine, the recommended phase II dose was 20 mg/m^2^. This concentration of PEV in combination with 75 mg/m^2^ of azacitidine led to no additional toxicities and yielded an 83% overall response rate in patients who received at least six cycles of therapy. Taken together, these studies have established that PEV is safe in humans, with an intriguing efficacy signal in AML.

No clinical trials specific to head and neck cancer patients have been initiated to date with PEV. However, head and neck cancer patients have received PEV as part of a variety of advanced solid tumor trials. Of particular interest is NCT01862328, an open-label, multicenter, phase 1b study evaluating the safety and efficacy of PEV in combination with a variety of standard of care agents [62]. All patients enrolled had progressive disease following relevant standard therapy. Arm 1 consisted of a PEV and docetaxel regimen. Intravenous PEV was administered at 15 mg/m^2^ on days 1, 3, and 5. Docetaxel was administered at 75 mg/m^2^ on day 1 of 21-day cycles. Of the 22 patients enrolled in arm 1, two were head and neck cancer patients with lung metastases. Both of these patients experienced a partial response (PR) as a result of treatment.

Arm 2a combined PEV with carboplatin therapy. PEV was administered at 15 mg/m^2^ on days 1, 3, and 5 in combination with carboplatin AUC6 on day 1 of a 21-day cycle. This lead-in cohort included a single head and neck cancer patient. This patient received three cycles of PEV and carboplatin and experienced a PR. Finally, arm 2 added 175 mg/m^2^ of paclitaxel on day 1 of 21-day cycles to the regimen administered in arm 2a. This arm demonstrated an overall response rate (ORR) of 35%, which included two of the three head and neck cancer patients enrolled. Both of these patients achieved a PR, and received 12 and 6 cycles of the therapy. The median duration of response for this arm was 5.9 months. All head and neck cancer patients enrolled in this study had already received at least one platinum-based chemotherapy. These results demonstrate that PEV could potentially have therapeutic benefit for head and neck cancer patients, particularly if administered before the tumor has developed resistance to standard of care genotoxic agents. The investigation of PEV in combination with cytotoxic agents in a head and neck cancer-specific trial could be justified.

### 3.2. TAS4464

TAS4464 (TAS) is a second-generation NAE inhibitor recently synthesized by Taiho Pharmaceuticals (Tokyo, Japan) [65]. TAS and PEV each have a sulfonamide group that is reported to form a covalent bond between the compound and NAE, thus impairing enzymatic activity. Similar to PEV, TAS was shown to cause a significant stabilization of CDT1 and p27. TAS displayed a high degree of cytotoxicity across a variety of cancer cell lines with particularly low half maximal inhibitory concentration (IC50) values in hematologic cell types. IC50 values for patient-derived cancer cells were between 0.2–4223 nmol/L. Cells treated with TAS displayed high levels of activated caspase-8 and caspase-9, signifying the activation of both the intrinsic and extrinsic apoptotic pathways [64]. TAS yielded significantly reduced tumor burden in the THP-1 xenograft model of AML as well as a patient derived xenograft (PDX) model of small cell lung cancer [64,65]. TAS has also been reported to display significant antitumor activity in a xenograft model of multiple myeloma (MM) when combined with various standard of care agents [66]. Together, these results demonstrate the potential of TAS for therapeutic use in a wide variety of cancer types.

To date, only one clinical trial has been initiated to investigate TAS in human patients. A phase I/II study to evaluate the pharmacokinetics, safety, and efficacy of TAS in MM and lymphoma patients began in March 2017 [67]. The study has since been terminated after enrolling 11 patients. Little information was given as to the cause of study cessation. With no new clinical trials currently planned or enrolling patients, the clinical development of TAS appears to have been put on hold.

### 3.3. ZM223

The first two compounds discussed are potent NAE inhibitors, which both form a covalent adduct in the enzymatic active site via a sulfonamide group. While this is the most well-documented mechanism of NAE inhibition, other compounds have been designed to block NEDDylation [74]. However, one challenge in designing these drugs is the similarity of NAE to other E1 ligases in the cell [75]. This has resulted in many synthesized NEDDylation inhibitors that also bind and impair ubiquitylation and SUMOylation [76,77]. For this review, we will focus on NEDDylation-specific inhibitors, as off-target impairment of other pathways is likely to impact the anticancer activity and toxicity of new drugs. ZM223 is a small molecule discovered through target-based screening that is thought to bind to the NAE active site without the presence of a sulfonamide group [78]. ZM223 displayed IC50 values of 0.1 μM and 1.22 μM in HCT-116 colorectal cancer cells and U-2 OS osteosarcoma cells, respectively. While ZM223 yields similar IC50 values to PEV in vitro, point mutations affecting the affinity of PEV to NAE have been shown to contribute to resistance; thus, compounds with different binding groups may play an important role in NEDDylation inhibition [68]. Further investigation is needed to determine if ZM223 can block NEDDylation in PEV-resistant cancer cells.

### 3.4. DI-591

The aforementioned compounds specifically targeted NAE, resulting in the inhibition of all NEDDylation events and the inactivation of all CRL family members. While NAE certainly appears to be the most druggable target in the NEDDylation cascade, downstream enzymes also present some opportunity for pharmacological inhibition. UBC12 is one of two NEDD8 E2 ligases that preferentially target CRLs for NEDDylation. This is accomplished through a regulated binding interaction within Defective Cullin NEDDylation 1 (DCN1) and a NEDD8 molecule, resulting in the transfer of NEDD8 from UBC12 to a CRL [79,80,81,82]. There are a total of five DCN family members; however, only DCN1 is well understood [83]. Multiple studies have found that DCN1 contributes to cell proliferation [84,85,86]. In addition, Sarkaria et al. demonstrated that the portion of chromosome 3q which harbors the DCN1 gene is often amplified in head and neck cancers [84]. This same study concluded that overexpression of DCN1 was oncogenic and allowed for transformation of HaCaT cells. Thus, there is sound rationale to design compounds affecting the interactions between UBC12 and DCN1, particularly in tumor types such as head and neck cancer.

DI-591 is a small molecule that disrupts the binding of UBC12 and DCN1, thus prohibiting conjugation of NEDD8 to CRLs [69]. DI-591 was designed to mimic the binding dynamics of a 12-residue portion of UBC12, which is responsible for the interaction with DCN1. Because of this, DI-591 forms a tight bond (K_i_ = 12 nM) with DCN1. Initially, disruption of UBC12–DCN1 activity was thought to specifically impair Cullin-1. However, it was shown that treatment with DI-591 in a variety of cancer cell lines resulted in the inhibition of Cullin-3 with little to no effect on any of the other CRL family members. While sub-micromolar concentrations of DI-591 were able to inhibit the NEDDylation of Cullin-3 in cancer cells, it did not display any cytotoxic properties in concentrations up to 20 μM. Due to these findings, DI-591 itself likely has little anticancer therapeutic potential, but may prove to be a valuable tool compound to study Cullin-3 activity in cells.

While this may eliminate DI-591 as a potential therapeutic, it does provide some interesting insights into how targeting NEDDylation affects cancer cell viability. It is possible that Cullin-3 is not essential for cancer cell proliferation and that its inhibition is not a major factor underlying the therapeutic benefit associated with the previously mentioned compounds. Furthermore, the lack of anticancer effects of DI-591 may also suggest that broad inhibition of the CRL family, or even the cell-wide blockage of NEDDylation, is needed to effectively kill cancer cells. If this is the case, compounds specifically targeting the upstream NAE may prove to be more potent and effective anticancer agents.

### 3.5. NAcM-OPT

NAcM-OPT is another small molecule compound synthesized to target the interaction of UBC12 and DCN1. NAcM-OPT was designed to interrupt the docking of the N-terminal acetylated methionine of UBC12 to its appropriate docking site on DCN1 [70]. Much like DI-591, NAcM-OPT binds to and blocks the deep, hydrophobic pocket to which the acetylated methionine of UBC12 would fit into. This mechanism disallows the proper binding interactions of the two proteins. This compound was shown to potently inhibit the binding of UBC12 and DCN1 with an IC50 value of 79 nM. In vitro analysis showed that NAcM-OPT caused a decrease in the NEDDylation levels of both Cullin-1 and Cullin-3 and prevented soft-agar colony formation of HCC95 cells, a lung cancer cell line with high baseline levels of DCN1. Thus, blocking the interaction of UBC12 and DCN1 did yield an anticancer effect in the case of this particular compound. The reasons behind the disparity in in vitro activity between DI-591 and NAcM-OPT is not fully understood, given the compounds similarity in proposed mechanism of action. In vivo work has shown that NAcM-OPT is orally bioavailable and has a half-life of 4.2 h in mice [71]. These data provide evidence that NAcM-OPT may have therapeutic value, particularly in cancers with elevated levels of DCN1. Given that DCN1 is commonly overexpressed in head and neck cancers, further investigation is warranted.

## 4. Conclusions

Despite advances in cytotoxic, targeted, and immunomodulatory cancer therapies in the last few decades, patients with locally advanced head and neck cancer have limited survival if surgical resection and RT are not curative. Currently, genotoxic agents, particularly cisplatin or platinum-based combinations, are the accepted standard in multimodality, curative treatment plans. The NEDDylation pathway mediates a specific post-translational modification that controls protein degradation. Key regulators of NEDDylation activity have been shown to be overexpressed in many forms of cancer, including head and neck cancer. The NEDDylation cascade relies on multiple enzymes to tightly regulate the process. To date, compounds inhibiting NAE and UBC12 activity have been developed. Of the compounds discussed in this review, PEV has the most solid preclinical foundation and has demonstrated significant potential in early phase clinical trials. While not all compounds discussed yield high anticancer activity, the body of evidence suggests that inhibitors of NEDDylation are worthy of further exploration in order to improve cancer patient outcomes.

## Figures and Tables

**Table 1:** Cullin-RING Ligases and Selected Targets.

CRL	Key Targets	References
Cullin-1	BIM-EL, p21, p27, Wee1	[33,38,39]
Cullin-2	HIF1α	[40]
Cullin-3	Nrf2, Beclin1, ULK1	[41,42]
Cullin-4A/4B	XPC, CDT1, p21	[43,44]
Cullin-5	NOXA, ATM	[45,46]
Cullin-7	Caspase-8	[47]
Cullin-9	Regulates p53 localization	[48]

Abbreviations: BIM-EL—Bcl-2-like protein 11 extra long; p21—cyclin-dependent kinase inhibitor 1; p27—cyclin-dependent kinase inhibitor 1b; Wee1—Wee1 G2 checkpoint kinase; HIF1α—hypoxia inducible factor 1 subunit alpha; Nrf2—nuclear factor erythroid 2-related factor 2; ULK1—serine/threonine-protein kinase ULK1; XPC—xeroderma pigmentosum group C protein; CDT1—chromatin licensing and DNA replication factor 1; NOXA—Phorbol-12-myristate-13-acetate-induced protein 1; ATM—ataxia-telangiectasia mutated; p53—tumor protein P53.

**Table 2:** Compounds Targeting NEDDylation.

Compound	Target	Development Stage	References
Pevonedistat (MLN4924)	NAE	Clinical Trial: Phase III (AML); Phase II (Advanced Solid Tumors)	[49,50,51,52,53,54,55,56,57,58,59,60,61,62,63]
TAS4464	NAE	Clinical Trial: Phase I/II (Multiple Myeloma, Lymphoma)	[64,65,66,67]
ZM223	NAE	Preclinical (Osteosarcoma)	[68]
DI-591	UBC12/DCN1	Preclinical—No Cytotoxicity	[69]
NAcM-OPT	UBC12/DCN1	Preclinical (Lung)	[70,71]

Abbreviations: NAE—NEDD8 activating enzyme; UBC12—NEDD8 conjugating enzyme Ubc12; DCN1—defective in Cullin NEDDylation 1; AML—acute myeloid leukemia.

## Abbreviations

HPV	Human Papillomavirus
RT	Radiation therapy
5-FU	Fluorouracil
mAb	Monoclonal antibody
PD-1	Programmed death receptor-1
RTOG	Radiation Therapy Oncology Group
NEDD8	Neural precursor cell expressed, developmentally-downregulated 8
UBA3	Ubiquitin-like modifier activating enzyme 3
APPBP1	NEDD8 activating enzyme E1 regulatory subunit
NAE	NEDD8 activating enzyme
UBC12	NEDD8 conjugating enzyme Ubc12
UBE2F	NEDD8 conjugating enzyme 2
RBX1	RING-box protein 1
RBX2	RING-box protein 2
COP9	Constitutive photomorphogenesis 9
CRL	Cullin RING ligase
CAND1	Cullin-associated NEDD8-dissociated protein 1
p21	Cyclin-dependent kinase inhibitor 1
p27	Cyclin-dependent kinase inhibitor 1b
Wee1	WEE1 G2 checkpoint kinase
RING	Really interesting new gene
Ub	Ubiquitin
PEV	Pevonedistat
CDT1	Chromatin licensing and DNA replication factor 1
PI-FACS	Propidium Iodide-fluorescent activated cell sorting
AML	Acute myeloid leukemia
MM	Multiple myeloma
HNSCC	Head and neck squamous cell carcinoma
NOXA	Phorbol-12-myristate-13-acetate-induced protein 1
BIM	Bcl-2-like protein 11
BH3	Bcl-2 homology 3 domain
TRAIL	Tumor necrosis factor-related apoptosis-inducing ligand
CDT2	Denticleless protein homolog
Gy	Gray (unit of radiation)
Chk1	Checkpoint kinase 1
Chk2	Checkpoint kinase 2
γH2AX	Gamma H2A histone family member X
PR	Partial response
AUC_6_	target area under the concentration-time curve of 6 mg/mL * min
ORR	Overall response rate
TAS	TAS4464
DCN1	Defective in Cullin NEDDylation 1
IC50	Half maximal inhibitory concentration
